# A hydrographic dataset of the Wadden Sea as a foundation for a digital twin of the coastal ocean

**DOI:** 10.1038/s41597-025-06211-1

**Published:** 2025-11-05

**Authors:** Robert Lepper, Markus Reinert, Jannek Gundlach, Julietta Weber, Frank Kösters

**Affiliations:** 1 Federal Waterways Engineering and Research Institute (BAW), Hamburg, Germany; 2 Federal Maritime and Hydrographic Agency (BSH), Hamburg, Germany

**Keywords:** Physical oceanography, Databases, Natural hazards

## Abstract

The Wadden Sea stretches along the coasts of the Netherlands, Germany, and Denmark in Western Europe. As the world’s largest coherent channel-shoal environment and as a UNESCO world heritage site, managing economic and political interests is complex. All stakeholders require a common understanding through shared, public databases and tools, e.g., to comply with the strict national park legislation. Although digital twins of the European oceans are emerging, current solutions still lack spatial resolution and neglect changes in coastal morphology. Here, we present a hydrographic dataset of the Wadden Sea covering the period of 2015 to 2022 with annually updated topography, and we outline a framework for the transition of digital twins toward the coastal oceans. A coupled numerical modeling approach was used to derive tides, currents, waves, salinity, temperature, and suspended sediments in 20-minute intervals. All data were published in an open-access governmental repository as files and web services with FAIR metadata documentation.

## Background & Summary

Digital twins are a virtual representation of a system or mechanism that mirrors a corresponding physical entity^1^. In the Earth sciences, it is a technology to converge big data from various sources about natural systems to meaningful digital information^1,2^. One of the main applications of a digital twin is to support sustainable governance, because precise and recent data are indispensable to make efficient decisions and to minimize risks^3^. In the Earth sciences, a digital framework qualifies as a twin when the conditions of bidirectional communication, dynamic interaction, and real-time connection between the digital and the physical world are met^2,4^. Consequently, the key to the evolution of process-based or artificial intelligence (AI) models toward digital twins lies in their connection with the broader public and in the continuous replication of past-, current- and future states.

Digital Twins of the Ocean (DTOs) are direct extensions of a Digital Twin Earth and emerged as a key technology in hind-, now- and forecasting by interactively integrating physical and virtual assets in and around the ocean. A DTO should include concepts of real-time display, dynamic process-understanding with the capability of prediction, and supporting utilities, e.g., information communication or a supported decision-chain^4,5^. The goal of the European DTO, for example, is making ocean knowledge readily available to citizens, entrepreneurs, scientists, and policy makers to support a resilient and sustainable blue economy as part of the European Union’s Green Deal initiative. Since roughly 2.15 billion of the world’s population live near a coast^6^, downscaling DTOs to the coastal ocean becomes inevitable, e.g., for coastal zone management or environmental assessments.

DTOs, however, lack resolution near the coasts and therefore cannot account for many three-dimensional shallow water processes, e.g., river plumes or regions of fresh water influence. Major known limitations of downscaling DTOs toward the coastal ocean are the adequate consideration of highly-dynamic and unforeseeable coastal morphodynamics^7–9^, sub-mesoscale estuarine dynamics, and intertidal wetting and drying, all of which are connected through direct or indirect feedback processes^8^. While there are concepts for downscaling DTO information toward the coastal ocean^10^, to our knowledge, no dynamic operational system has been established in the European seas, yet. In addition, systematic long-term hydrographic surveys of coastal systems are sparse; only local datasets exist with limited spatial and temporal coverage, e.g., for the French Loire estuary^11^ or the German Bight^12^. Examples of digital twin like studies in coastal oceans include only specialized applications such as monitoring of maritime vessel operations^13^, the development of nature-based solutions using sea-grass^14^, coastal flooding monitoring^15^, or disaster response coordination^16^. None of the above implemented a holistic coastal ocean, instead, they are restricted to specific applications and are spatially and temporally limited.

Here, we present a concept for bridging the gap between DTOs and the coastal ocean by using a prototype of a digital twin of the coastal ocean at the Wadden Sea (North Sea, Western Europe) and provide data in the period of 2015 to 2022. The paper is structured as follows: We outline a pathway toward digital twins of the coastal ocean (1), present all published data for the period of 2015 to 2022 and their availability (2), conduct a technical validation (3), and demonstrate practical value by showcasing best-practice use cases (4).

## Methods

### Digital twin framework

**Fig. 1:**
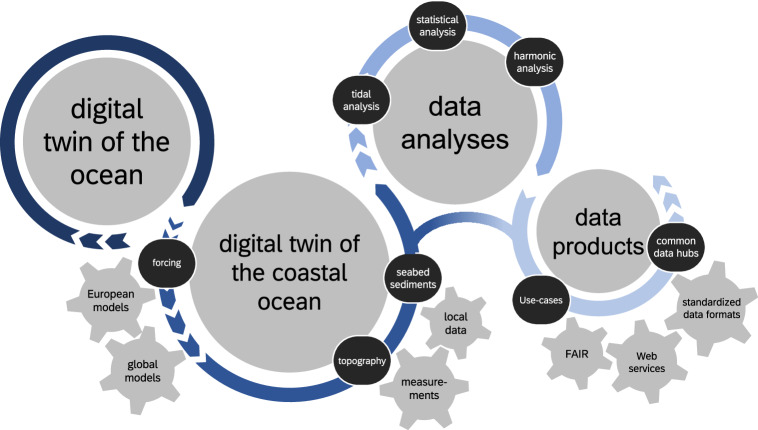
Workflow for downscaling global and regional information from digital twins of the ocean (DTO) to coast-related information using a digital twin of the coastal ocean (DTC). Data from a DTO were used to force the DTC and measurements and local data were used to update the model’s physical assets. Modeling results were processed to characteristic annual parameters. Finally, model and analyses outputs were standardized to data products, published FAIR and distributed using common web services.

To elevate a conventional model toward a DTO, the following three conditions must be met: (1) real-time connection, (2) bi-directionality, and (3) interactivity^2,4,5^. For distinguishing a digital twin of the coastal ocean (DTC) from a DTO, we recommend to add the criterion (4) *downscaling*, ensuring a clear link to the DTO and a concept for considering local geometric properties and their changes over time appropriately. We chose the following combination of processing steps for passing information from global and regional models and DTOs to the coastal ocean (Fig. 1).

At the heart of our prototype DTC lies a process-based, three-dimensional, numerical model as a virtual representation of our coastal system. First, forcing data were extracted from the European operational data sets, from European meteorological and river runoff data, and from a global tide model at the open boundaries of the model. Second, local coastal features, i.e., recent topography and seabed sediment from observational data, were interpolated onto the computational grid to represent the system’s physical properties. Third, a simulation was conducted to derive physical parameters, e.g., sea surface elevations, salinity, and wave properties. In our approach, we added tidal, statistical, and harmonic analyses to reduce the simulation outcome to characteristic parameters. Finally, a web-environment unifies all data and facilitates user interaction. To ensure best-practice, use cases of our data were linked to the metadata, which is common practice in similar data publications, e.g., the European Copernicus services. Complying with standardized interoperable metadata formats, i.e., FAIR, ensures our data’s availability throughout national and international hubs.

In the following, we compare the DTC framework to the conditions for a DTO:

1. **Real-time connection:** All steps in our current framework (Fig. 1) must still be conducted in a hindcast simulation, because topography and seabed sediments observations are required input parameters for a process-based coastal model. Therefore, an inevitable time-lag exists, until these crucial parameters become available, which contrasts the “real-time connection” condition of DTOs. This is a current limitation on the pathway to a full DTC that can be applied anywhere in the world, except for morphologically stable coasts. One promising way forward to bridge this gap in the future present remote-sensing topographies^17^ or other data-driven topography extrapolation^7^. As the effort involved in the implementation and validation of such an approach is still unclear, we refer this extension to future research.
2. **Bi-directionality**: This condition was satisfied by co-designing data products in practical use cases in and around the Wadden Sea with potential users. Use cases are, e.g., the estimation of habitats using tidal parameter overlaps, cable routing based on perennial topography, identification of annual intertidal zone topography as an intersect between topography and modeled tidal high and low water, or extracting the local speed of tidal propagation for planning LiDAR campaigns.
3. **Interactivity:** We made a first step toward a modular, interactive component by developing a web-viewing module with the possibility of adding user-defined web services or by incorporating web-GIS features such as dynamic differences, time-series extraction, or dynamic data cropping. In addition, we set up standardized web services that enable similar implementations in any other data hub.
4. **Downscaling:** Annually updated topographies and boundary conditions from the European DTO and global tide models were considered in a high-resolution regional model to ensure that local features were represented. This criterion could be improved by closing the gap toward an operational window as described under (1) real-time connection.

### The Wadden Sea

**Fig. 2:**
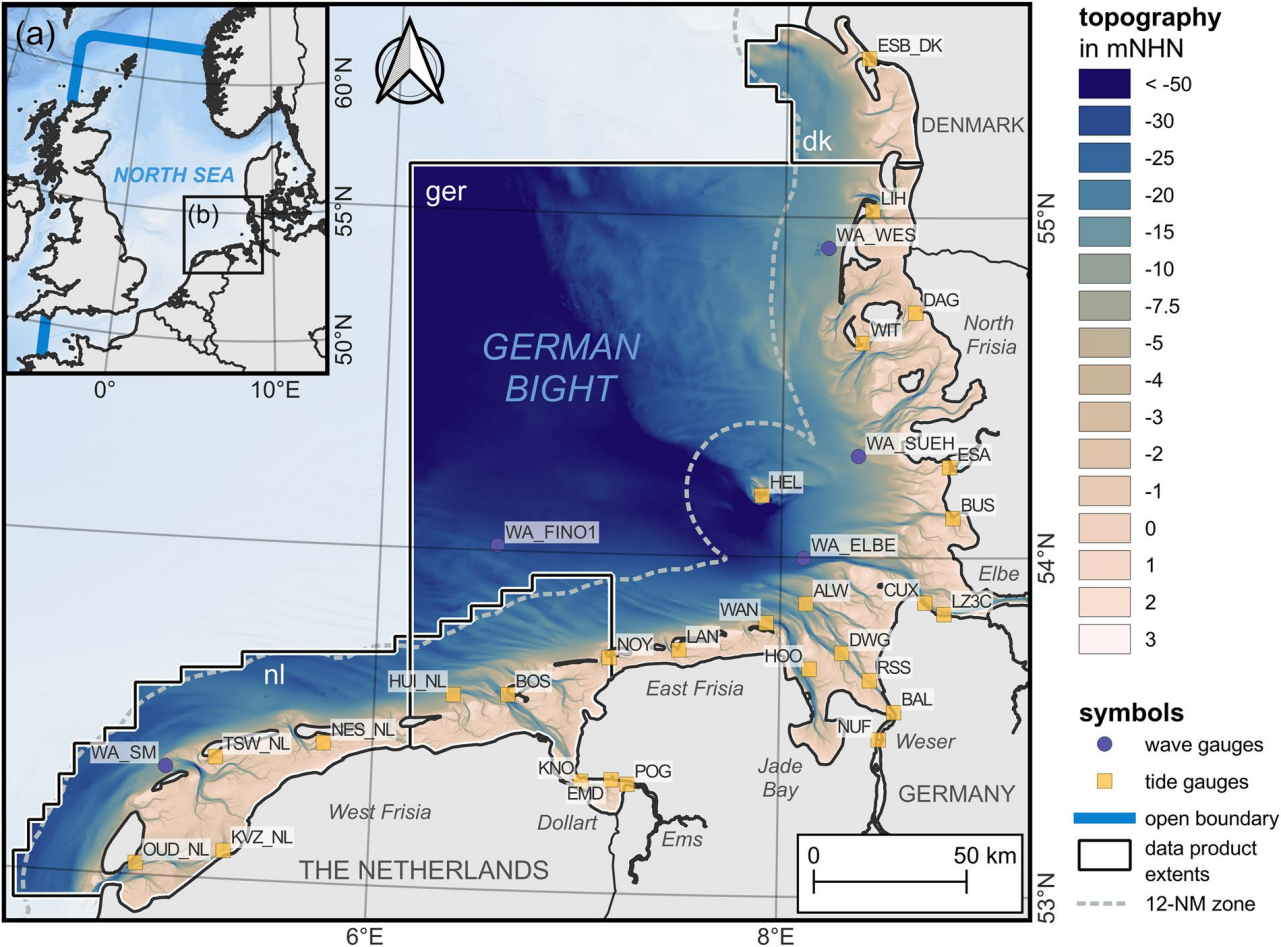
Map of the numerical model (**a**) with the model’s open boundaries in light blue and the focus area in a grey box (**b**). The focus area in (**b**) includes the Wadden Sea from the Netherlands in the Southwest to Germany and Denmark to the Northeast. Blue dots indicate wave measurements and orange markers indicate sea surface height, current velocity, sea water temperature, and sea water salinity gauges. Light grey patches represent land and the light blue shades in (**a**) and **(b**) are part of the EMODnet (2022) digital terrain model. NHN refers to German chart datum and NM denotes nautical miles.

Our study area is the Wadden Sea in the southeastern North Sea on the European Continental Shelf (Fig. 2). It is the world’s largest coherent channel-shoal system with a coastline of roughly 450 km. Its coastal zone consists of broad, poorly-vegetated, or bare tidal flats breached by deep channels, several small and large estuaries, barrier islands, islands, tidal inlets, salt marshes, and sandy shoals with shallow embankments. The Wadden Sea has been a UNESCO world heritage site since 2009, protected for its unique biodiversity and for being one of the last remaining natural landscapes in Europe. Tidal range is mesotidal, waves can reach a fully-arisen state, and the storm season starts in September and ends in April.

Despite the presence of highly frequented shipping routes, active coastal zone management, frequent scientific research, unique habitats, and extensive recreational usage, no shared international database or digital twins of this area exist to our knowledge. Several data collections covered the North Sea, e.g., the CoastDat2 (1.8 km grids) project (Geyer, 2014; Groll and Weisse, 2017) or the Copernicus physics re-analysis on coarser grids (European Union - Copernicus Marine Service, 2020). A higher-resolution data collection exists for the German Bight for the period of 1996 to 2015 (Hagen *et al*., 2021b; Sievers *et al*., 2021), including topography, surface sediments, tides, salinity, and waves. Yet, even these data are provided on coarse grids for coastal application, i.e., 1 km, and are furthermore outdated as the last entry dates to the end of the year 2015. Additionally, they are spatially confined to the German exclusive economic zone.

### Observational data for model validation

Observational data in the Wadden Sea are collected through a dense system of gauges and wave riders operated by federal and local agencies. We chose a representative number of observations with a good spatial coverage to validate our model (Fig. 2). All observational data were checked semi-automatically for valid ranges and visually whenever implausible deviations to the model were evident. Implausible samples were flagged and discarded after review. Observational sea surface elevations have a temporal resolution of 1 minute and sea water temperature, salinity, and suspended sediment concentration (SSC) time-series were provided in regular 10-minute intervals. Wave observations were often non-equidistant which is why they were unified to regular 30-minute intervals. It should be noted that the wave observations were subject to frequent data gaps or implausible data. SSC time-series in the outer Weser were converted from turbidity observations using a water-sample-calibrated conversion function^18^. The very high sediment concentrations, i.e., fluid mud, in the Ems estuary cannot be captured by our model, which is why a comparison there could be misleading. In addition to observed time-series, the suspended sediment concentration from roughly 1,300 water samples (excluding organic material) were compared to the model. Data for the outer Weser were adapted from the German Federal Waterways Engineering and Research Institute (BAW), data for the German Bight were adapted from the Federal Maritime and Hydrographic Agency (BSH)^19^, and data for the Dutch Wadden Sea from Rijkswaterstaat.

### Hydrodynamic model

Model set-up and software were adapted from previous work^12^; only a brief summary of the technical updates is presented here (Fig. 2a). We used the hydrodynamic, process-based UnTRIM² (unstructured, tidal, residual, intertidal, mudflat model) modeling framework^20,21^, the SediMorph sediment transport module^22^, and the unstructured k-model for spectral wave modeling^23^. The model domain extends from the tidal weirs of the estuaries over the Wadden Sea to Norway, Scotland, into the English Channel and to the Belt in the Baltic Sea (Fig. 2a). Computations were conducted annually in the period of 2015 to 2022. Initial sea surface height (SSH) and current velocity were taken from a predecessor year. Initial salinity and sea water temperature in the spin-up were nested from the three-dimensional daily Copernicus Global Ocean Physics Analysis and Forecast (https://data.marine.copernicus.eu/). Suspended sediments were initialized from a constant low concentration, and the wave model computes initial spectra from the respective wind forcing. All simulations use a three-month model spin-up period.

The horizontal grid resolution consists of 342,000 unstructured cells with 541,000 interfaces to achieve a resolution between 200 and 600 m in the coastal zone of the Dutch, German, and Danish Wadden Sea. Roughly 70% of all grid cells were placed in the coastal zone of the Wadden Sea. Using the well-established subgrid approach of UnTRIM^2^ ^21^, this computational grid was refined by a factor of 3 in the entire model domain, by a factor of 7 in the 12- nautical mile zone of the Wadden Sea, and by a factor of 10 in the German estuaries Ems, Weser, Eider, and Elbe to obtain a total of more than 16,000,000 subgrid cells for an accurate representation of the complex wetting and drying topography. The chosen vertical resolution ranged from 0.5 m near the sea surface (4 to −4 mNHN, with NHN representing German chart datum), to 1 m in the subtidal coastal zone and −20 mNHN gradually becoming coarser downwards. The grid covers floodable low-lying terrain (e.g., Hallig marshes) and shoals to allow submergence during storm surges. Training walls, groins, large breakwaters, dikes, barrages, and other relevant structures were incorporated in the model’s subgrid resolution by adjusting the flow section between computational elements. Major storm surge barrages in the Eider (closed at +1.8 mNHN) and Ems (closed at +3.6 mNHN) estuaries were incorporated. The topography in our focus area was updated annually^7,24^ and the remaining areas were filled using the 2022 EMODnet digital terrain model^25^ with heights relative to mean sea level (MSL). Surface sediments were derived from a mixed modeling-measurement approach in the Wadden Sea^26,27^ and the remaining areas consist of linearly-interpolated, observed sediment samples obtained from European authorities. Bottom roughness of the model was estimated using a bedform predictor^28^ and adjusted in model calibration by applying local additional roughness, e.g., in the inner estuaries. Sea surface elevations at the open boundary were reconstructed from global FES 2014b tidal constituents^29^, and modified with the inverse barometer correction for surge^30^. To correct for the remaining bias, observational sea surface elevations of the gauges Helgoland (Germany, northern boundary) and Roscoff (France, southern boundary) were used to match MSL, seasonal, and shorter-term fluctuations^12^. Three-dimensional salinity and sea water temperature at the open boundary to the North Atlantic were adapted from the Copernicus^31^ ocean physics reanalysis. The atmosphere-ocean interaction uses a composite heat-flux approach with wind speed, atmospheric pressure, surface temperature, solar radiation, humidity, and cloudiness adapted from reanalyzed COSMO-REA6 until 2018^32^ and from ICON-EU (2019 to present, https://dwd-geoportal.de/products/G_D5M/). River runoff was considered whenever the long-term average runoff was larger than 100 m^3^/s; we used a Dirichlet flow boundary in this case. A total of 37 freshwater runoffs were obtained from the German Water and Shipping Administration (available on request), from Rijkswaterstaat (https://waterinfo.rws.nl/), and from the Copernicus Marine service (https://marineinsitu.eu/). Data outside of the focus area, apart from the Rhine-Meuse-Scheldt delta, were averaged to their long-term monthly means over the maximum length of available records. The SSC of all rivers was adapted from Zwolsman (1994)^33^.

### Wave model

The spectral wave model was run on a separate unstructured grid based on the hydrodynamic grid (not included). To reduce computational cost, the grid was limited to the German, Dutch, and parts of the Danish exclusive economic zones. Estuaries were neglected. The grid resolution of the wave model is identical to the hydrodynamic model near the coast; transitional areas in the southern North Sea (starting at the −30 mNHN height isobath) consist of coarser rectangular elements. Wave spectra were resolved at 32 frequencies (0.006 to 1.6 Hz) with 24 directions, i.e., 15° steps. UnTRIM² and the unstructured k-model (UnK) were run coupled by exchanging sea surface elevation, current velocity, meteorological forcing, wave properties, and wave radiation stresses at every time step. Wave-induced shear stress was used in SediMorph to consider wave-driven erosion. Basic wave physics such as refraction, reflection, and diffraction are represented in the model but the UnK physics engine is currently unable to consider wave-wave interaction (triads, quads), white-capping, and wave breaking. Nevertheless, the model produced realistic results in shallow domains such as the southern North Sea in the past^12^. The wave model’s main purpose was to recreate wave-current interaction and to induce wave-induced shear stress for sediment transport. However, it must be noted that wave data from our digital twin underly limitations as presented in the subsequent technical validation. The wave-module is scheduled for a thorough update to mitigate this limitation in subsequent data.

### Sediment transport parametrization

Grain size distributions from sediment samples were divided into discrete sediment classes (Table 1) and then interpolated to the computational grid. Due to a lack of spatial coverage in the Ems and Weser estuaries, simplified, uniform sediment distributions were assumed for model spin-up using fine fractions in the Ems and in the estuarine maximum turbidity zone (ETM) of the Weser estuary. We adapted a simple definition for the suspended sediment fractions in the model by distinguishing between very fine sediment particles (cohesive fraction 1, C1) with low settling velocity and their aggregates (cohesive fraction 2, C2) with a higher sinking velocity^34^. This choice was within the range of sinking velocity measurements in the German Bight^35^. In addition, the model moves very fine sand either as suspended load during high-energy, or as bedload during low-energy conditions. Erosional and depositional fluxes for the suspended load were computed using the Partheniades (1965)^36^ formulation and bedload fractions based on the van Rijn *et al*.^37^ transport equations. Flocculation of the cohesive fractions C1 and C2^38^ was allowed and hiding and exposure was considered^39^. The bed model was based on the common active layer concept^40^ with unified vertical discretization and grain size distribution^41,42^ as explained by Fricke and Malcherek (2014)^43^. The active layer thickness was chosen at 10 cm and all underlayers had an initial thickness of 2.9 m; a total soil thickness was therefore 3 m.

**Table 1:** Sediment fractions for the sediment transport models and the respective transport mode and parameters with w_s_ being the sinking velocity and τ_krit,e_ being the critical bed shear stress for erosion.

Fraction name	Transport mode	Median diameter	w_s_ in mm/s	τ_krit,e_ in N/m^2^
C1	suspended	20 µm	0.25	0.15
C2	suspended	60 µm	1.5	0.15
very fine sand	suspended/bedload	90 µm	7.8	—
fine sand	bedload	175 µm	—	—
medium sand	bedload	350 µm	—	—
coarse sand	bedload	750 µm	—	—
very coarse sand	bedload	1500 µm	—	—

External sediment sources were considered at the open boundary of the model (constant 20 mg/l) off the British coast near Holderness, Norfolk and Suffolk (each ~1 to 2 Mt a^−1^), in the English Channel (~50 Mt a^−1^) and off the Scottish coast near Moray Firth at Flemish Banks (~30 Mt a^−1^) based on literature^44,45^. Since most external sediment sources are believed to result from cliff and shore erosion during stormy conditions^46,47^, a method was followed to increase the sediment input at this time^48^. Human activity, i.e., nourishments or dredging and dumping, were not incorporated in the model. Most dredging and dumping operations are confined to estuaries with considerable amounts of relocated material being re-imported. We believe that, given the spatial scale of the model, local human activities may be neglected if the model neglects morphodynamics. Nevertheless, we recognize that they should be a future component of the model and the digital twin framework.

The effects of advanced sand-mud interaction^49^, consolidation, biological feedback, sea grass, and fluid mud phenomena were not yet incorporated in the model.

### Analyses

We conducted data analyses to reduce the complex and large output of the numerical model to annual characteristic parameters. We distinguish three kinds of analyses: (1) tidal characteristics, (2) harmonic, and (3) statistical analyses. All analyses reduce 20-minute interval model outputs from 26,280 output time steps per year (26,352 in leap years) to one tidal constituent’s amplitude and phase, to 704–706 tides, or to one statistical parameter (e.g., a quantile). The analyses were conducted with the access-restricted NCANALYSE framework (https://wiki.baw.de/en/index.php/NCANALYSE, last access July 10 2025), which extends a classic Eulerian analysis with a Lagrange-like technique where a tide at each point is linked to a predecessor leading to an identical number of tides across the model domain. We refer to Hagen *et al*.^12^ for further details. From 704–706 tides, averages or quantiles were calculated to obtain a representative impression of the parameter for each year. A harmonic analysis is a well-established procedure with sea surface elevations and is, e.g., described by Codiga (2011)^50^. Nodal modulation was not considered in our harmonic analyses as the commonly applied f-u correction is not valid at our study site^51^.

## Data Records

### General information and data availability

Modeling was conducted year-wise in the period of 2015 to 2022; therefore, all model and tidal analyses data were also created annually. The spatial extent covered by the data (Fig. 2b) was limited roughly to the 12 nautical-mile-zone (12-NM) off the coasts of the Netherlands and Denmark as well as to an area in the German Bight for which a data collection already exists^12^. For the latter, temporal data coverage was extended to a total of 27 years (1996 to 2022).

All datasets were split into three subsets according to authority (nl, ger, dk in Fig. 2) to limit file size. The unstructured model data were gridded to enhance usability and facilitate web-handling. The Dutch and German data overlap in the outer Ems to mitigate the need for data merging in this frequently investigated area. The grid resolution of model output (500 m), data analyses (20 m), topography (10 m), and surface sediment data (10 m) were set as a multiple of 10 with mutual coordinate origin so that an intersect remains possible without interpolation.

Simulation and analyses data from the digital twin, topography, surface sediments, and use cases are permanently stored in the data repository of the German Federal Waterways Engineering and Research Institute (BAW, https://datenrepository.baw.de/). In addition, web map services (WMS), web feature services (WFS), and web coverage services (WCS) were created when applicable. The web-viewing module can be reached under https://mdi-dienste.baw.de/viewer/. Metadata were structured by the following guiding themes and can be found by adding the keyword “digital twin” to the search window or by following the digital object identifier (DOI) of the following citations (e.g., digital twin topography):

- topography^24^,
- intertidal topography^52^,
- surface sediments^27^,
- hydrodynamic data^53^,
- data analysis, e.g., tidal analysis^54^,
- and use cases^55^.

All metadata are interoperable, linked to their respective web services as well as to best-practice recommendations from use cases, and can be found in German and European data hubs. In the following section, we elaborate on the extent of the guiding themes *hydrodynamic data* and *data analyses* as direct data outcomes from our DTC. Documentation and assessments of the topography^7^ and surface sediment data^26^ were published externally.

### Hydrodynamic data

Hydrodynamic data, i.e., the model output, was gridded to approximately 500 m and stored in a netCDF format in 20-minute intervals. Each file covers one full year from January 1 00:00 UTC until December 31 23:40 UTC. The provided coordinates are longitude and latitude (EPSG: 4326), the height reference system is NHN (i.e., German chart datum), which is equivalent to NAP (i.e., Dutch chart datum), and the time zone is UTC. To limit file size, we split the data for each year into four groups: Tides, transport, bed shear stress, and waves.

**Fig. 3:**
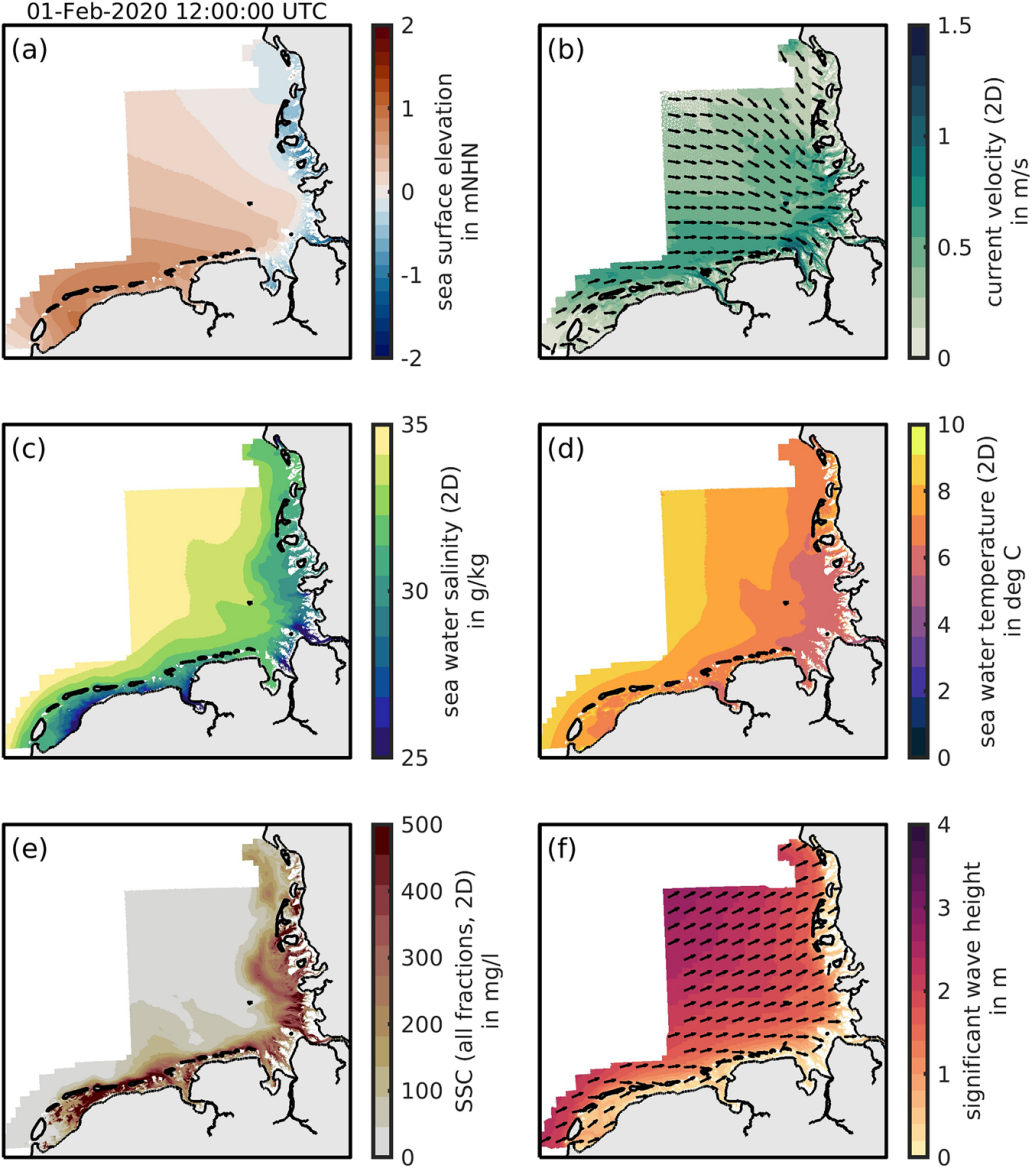
Exemplary modeling data on February 1 2020, 12:00 UTC (**a–f**), in the data coverage area from Fig. 2b. Vectors (**b,f**) were simplified to a uniform length and (**e**) suspended sediment concentrations (SSC) were converted to mg/l for this figure only. White patches represent no-data (i.e., no coverage) and light grey patches land.

Variable names and their attributes were inspired by the standardized UGRID conventions and the CF standard names (https://cfconventions.org/). Note that some parameters in Table 2 do not have CF-conform standard names or meaningful equivalents, yet. In this case, we resorted to common terms found in the literature (e.g., suspended sediment concentration). Three-dimensional parameters are referred to as depth-averaged (“_2d”), at the “_top”, and as “_bottom” layer of the numerical model. Vector data are denoted by x (eastward) and y (northward). Table 2 gives a full parameter list and Fig. 3 illustrates one exemplary time-step in February 2020.

**Table 2:** Overview of hydrodynamic data.

Parameter	variable name	Group
Sea surface elevation	sea_surface_height_2d	tides
Current velocity	current_velocity_2d current_velocity_2d_x current_velocity_2d_y	tides
Sea water salinity	sea_water_salinity_2d sea_water_salinity_top sea_water_salinity_bottom	transport
Sea water temperature	sea_water_temperature_2d sea_water_temperature_top sea_water_temperature_bottom	transport
Suspended sediment concentration	suspended_sediment_concentration_2d suspended_sediment_concentration_top suspended_sediment_concentration_bottom	transport
Effective bed shear stress	effective_bed_shear_stress_x_2d effective_bed_shear_stress_y_2d	shear_stress
Wave height	significant_wave_height	waves
Wave period	wave_mean_period wave_peak_period wave_period_tm01 wave_period_tm02	waves
Wave direction	wave_directional_spread wave_to_direction_x wave_to_direction_y	waves

All parameters are available for each year in the period of 2015 to 2022 in equidistant 20-minute intervals. Files are distinguished by year, group, and region, e.g., tides of the year 2020 in Germany are denoted as “tides_2020_ger.nc”.

### Data analyses

**Fig. 4:**
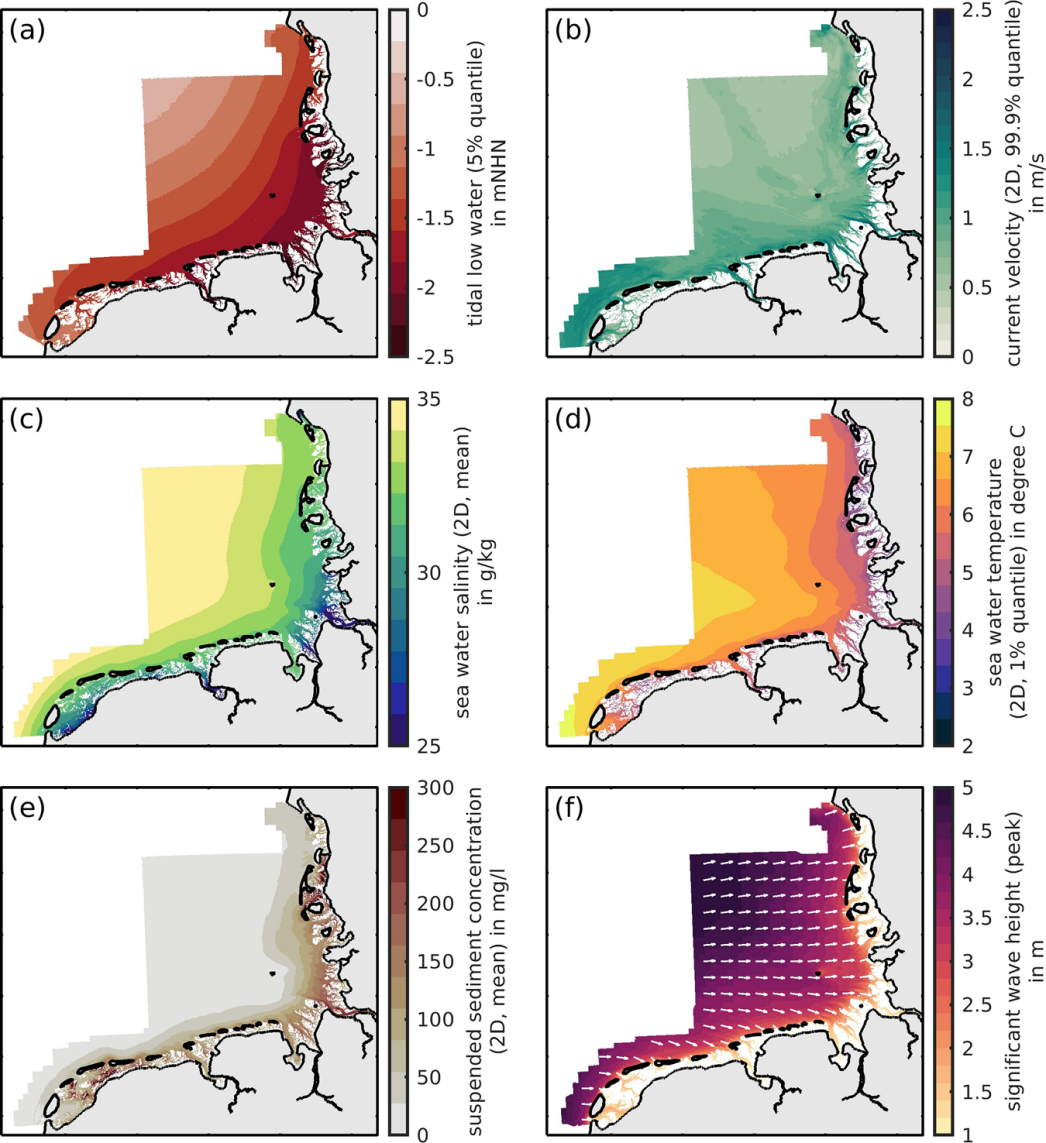
A selection of modeled analyses results of the year 2020 (**a–f**). Modeling outputs were reduced to quantiles of tides (**a**) or quantiles of all simulation time steps (**b–f**). the vectors in (**f**) represent the residual wave direction of the analysis period. All wetting and drying samples were filtered to ensure comparability. White patches represent no-data values and grey patches land.

Tidal and statistical analyses condense modeled time-series by deriving characteristic parameters. We chose to unify the long time-series of modeled data to annual characteristic parameters because we believe that they add value for the broad public and serve as low-level entry point to this complex dataset. All analyses were conducted on an entire year of numerical simulations, i.e., approx. 704–706 tides derived from 20-minute intervals. All analyses results were gridded to 20 m GeoTIFF files and come with a color vision deficiency compliant style (Fig. 4). Note that Fig. 4 only represents a selection of all provided analyses parameters. A full set of annual analyses consists of 62 parameters per year (Table 3) all of which were split into three regions (see Fig. 2). Averages, quantiles, and parameters were chosen based on the author’s experience and their work on the collaborative use cases.

**Table 3:** Overview of annual analyses parameters.

Parameter	File name	Variants	No filter version
M_2_ tidal constituent amplitude and phase	constituent_m2	A, g	no
Tidal high water	high_water	5q, median, 95q	yes
Number of tidal high water events	nof_high_water_events	—	no
Tidal low water	low_water	5q, median, 95q	no
Tidal range	tidal_range	5q, median, 95q	no
Tide mean water	mean_tide_water	50q	no
Tidal high water time delta to reference	high_water_time_delta_to_ref	mean	yes
Number of valid data points	nof_valid_data_points	—	no
Sea surface height	sea_surface_height	peak	yes
Sea surface height	sea_surface_height	min, mean, 1q, median, 99q	no
Current velocity	current_velocity	mean	yes
Current velocity	current_velocity	99q, 99.9q	no
Current velocity	current_velocity_residual	northward, eastward, magnitude	yes
Cubed current velocity	cubed_current_velocity	mean	yes
Bed shear stress	bed_shear_stress	99q	no
Bed shear stress	bed_shear_stress	mean	yes
Sea water salinity	sea_water_salinity	1q, 99q	no
Sea water salinity	sea_water_salinity	mean	yes
Sea water temperature	sea_water_temperature	1q, 99q	no
Sea water temperature	sea_water_temperature	mean	yes
Suspended sediment concentration	suspended_sediment_concentration	min, mean, peak	yes
Suspended sediment concentration	suspended_sediment_concentration	1q, 99q	no
Significant wave height	significant_wave_height	95q, 99q	no
Significant wave height	significant_wave_height	mean, peak	yes
Wave period (at max sig. wave height)	wave_mean_period_at_peak_hs	mean	yes
Residual wave direction	wave_residual_direction	—	yes

All parameters are available for each year in the period of 2015 to 2022. Different variants are distinguished by averages, minima, maxima, and quantile configurations. Files are denoted by file name and variant, e.g., the median tidal high water of the year 2020 is denoted as “high_water_50q_2020.tif”.

Many of the parameters in Table 3 are affected by wetting and drying on the intertidal flats of the Wadden Sea and consequently prone to misleading interpretations: For example, if a computational element is always dry during a simulation except for storm surge conditions, its median high water is much higher than at a nearby permanently inundated point because the median then results from storm surge sea surface elevations only. We applied a dry mask filter to mitigate this effect. The dry mask was based on the models subgrid topology which is typically seven times finer than the model’s grid resolution, i.e., roughly 20 to 40 m. Subgrid elements that were dry at any time during the computation were flagged for wetting, and drying influence and discarded in postprocessing.

Still, in several applications, e.g., habitat classification in the intertidal zone, information is needed despite potentially biased averages. We therefore created a version without the dry mask, denoted by “no_filter”, for tidal high water quantiles, for peak sea surface elevation, and for all mean conditions. We emphasize that “no_filter” data should always be interpreted in context with the number of valid samples or the number of valid tides, respectively. This information was supplied alongside the data collection and should be applied to avoid misleading conclusions based on potentially skewed averages.

### Data overview

It was necessary to conduct several analyses in model validation due to the long time series and the large number of parameters in the dataset. Metrics for sea surface elevation and waves are well established: Tides were compared against measurements, using the M_2_ tidal constituent amplitude and phase from harmonic analyses. The root mean square error (RMSE) of the M_2_ amplitude and phase was estimated over a selection of tidal gauges indicated in Figs. 2, 6. Sea surface elevations were further validated using tidal range for amplitude, high quantiles of tidal high waters for storm surge elevations, and flood duration for tidal asymmetry. The wave model was assessed with a conventional RMSE for spectral wave parameters. As wave observations suffer from outliers and data gaps, the annual completeness of the significant wave height observation was indicated in the validation. Salinity and sea water temperature were also compared using a RMSE metric following Hagen *et al*.^12^. SSCs were compared on varying, relevant timescales following the suggestions of Winterwerp *et al*.^56^: We decomposed the discrete SSC time series using a low-pass filter with a respective duration of 12.4 hours (semidiurnal tides), 14.6 days (spring-neap), 3 months (seasonal), and 1 year (annual) and used the modified Kling-Gupta efficiency (KGE’) to estimate model skill. KGE’ combines Pearson correlation, prediction error variability, and prediction bias^57,58^ and is commonly applied in hydrology because it can reproduce the variability and timing of observed data. The score has a range from −∞ to 1, with 1 indicating a perfect match. Knoben *et al*.^59^ state a threshold of >1−√2 as acceptable for mean flow conditions in hydrology although the specific context of the application must be considered. Generally, all KGE > −0.41 were considered fair in the following visual comparison.

## Technical Validation

### Tidal dynamics

One key objective of our DTC was to accurately represent tidal dynamics with the model, as they dominate currents, shape the coast by contributing to sediment transport, and cause large areas to fall dry twice a day. We compared the simulated sea surface elevation against observations at tide gauges (Fig. 2). Gauges were selected to achieve an overall good spatial coverage of study area, to include different local settings, i.e., coastal, offshore, and estuarine conditions, and to provide fair data completeness.

The sea surface elevation validation was split into three stages: First, we conducted harmonic analyses and computed the RMSE between model and measurement of the predominant M2 amplitude and phase at all stations. Second, we calculated the RMSEs of the tidal characteristics from 704 to 706 tides per year to quantify the agreement of the modeled signal with observations. In the latter, we used the RMSEs of the tidal range to validate tidal amplitude and flood duration for tidal asymmetry and shallow water effects. Third, we assessed the storm surge elevations by comparing model bias at the highest one percent of observed tidal high waters, i.e., roughly the 7 to 8 highest annual tidal high water events. Generally, model deviations of less than 10% from the observed value were considered as acceptable^12^; for example, sufficient skill was assumed when the RMSE of the M_2_ amplitude was below 12.5 cm (the typical M_2_ amplitude is between 1 and 1.5 m) or when the RMSE of the tidal range was below 14 and 38 cm.

**Fig. 5:**
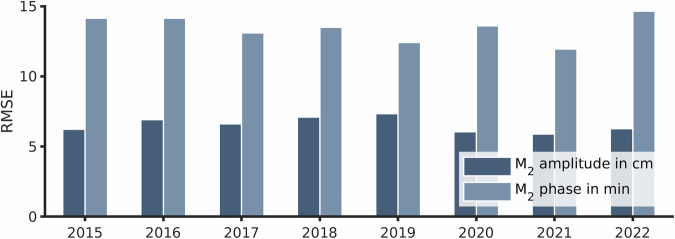
RMSEs of the M_2_ tidal constituent amplitude (dark blue shades) and phase (light blue shades) differences in the period of 2015 to 2022 for 20 representative tide gauges (compare Fig. 6).

The RMSE of the M_2_ amplitude varied between 5 and 7 cm across all years (Fig. 5). The best agreement was achieved in the period of 2020 to 2022 while the years 2018 and 2019 show slightly higher RMSEs in M_2_ amplitude. Spatially, the amplitude agreed well in the Dutch Wadden Sea and the outer Ems with errors of ± 5 cm; in the southern German Bight, the model slightly overestimated M_2_ amplitude by 2 to 10 cm. The RMSE of the M_2_ phase lag also showed comparable agreement across the entire period between 12 and 15 minutes, i.e., roughly 5°. It should be noted that phase lag was highest at the Dutch gauges Oudeschild (OUD) and Kornwerderzand (KVZ) while most other gauges varied only between 0 and 7° in offset (station-by-station assessment not included).

**Fig. 6:**
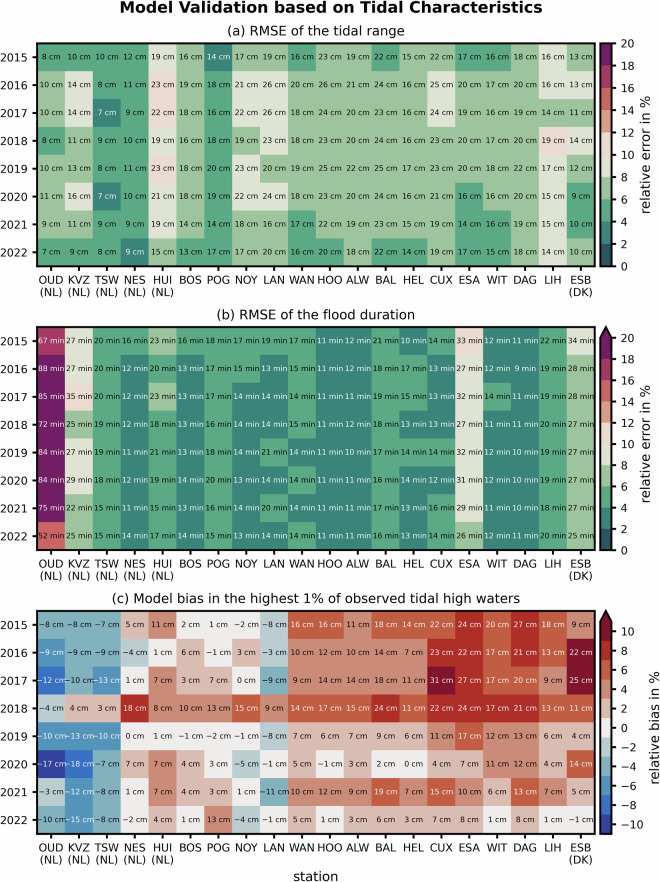
Validation of the sea surface elevation using the tidal range RMSE for tidal amplitude (**a**), the flood duration RMSE for tidal asymmetry (**b**), and the bias of the highest tidal high water events for storm surge elevations (**c**) at observational gauges in the data product area (see Fig. 2). Note that the relative error compares the error magnitude with the mean tidal range, amplitude, or high water level of the respective year and tide gauge.

According to the reasonable M_2_ amplitude agreement, tidal range was well represented by our model. The RMSE of all tidal ranges within a year were almost always less than 10% of the observed values, in some cases less than 4%. Only 5 out of 160 station-years exceeded a relative error of 10% (Fig. 6a). The best agreement was observed in the Dutch Wadden Sea with model deviations usually below 10 cm in RMSE. Considering that the typical tidal range is about twice as large at Bremerhaven (BAL), model errors of around 20 cm indicated a comparably good agreement with observations. Regarding the mean duration of the flood phase, good relative model agreement was found, with one exception at OUD (Fig. 6b).

At most stations, the RMSE of the flood duration was off the observed value by 10 to 20 minutes, corresponding to a relative error of about roughly 5%. We found errors exceeding 1 hour, i.e., up to 24% relative RMSE, only at the OUD tide gauge, which is likely related to the mixed diurnal-semidiurnal tidal signal at this station with an occasional small local maximum just before tidal high water. This phenomenon was not fully reproduced by our model and cannot always be captured by the tidal analysis method which relies on a distinct semidiurnal signal. This complies with a higher phase lag of the M_2_ tidal constituent at this location (approx. 11°, not included).

Modeled extreme tidal high water events, i.e., storm surges, improved after the switch in atmospheric forcing to the ICON-EU model in the year 2019 (Fig. 6c). In the period of 2015 to 2018, the model bias of extreme events can exceed 20 cm in the eastern part of the Wadden Sea. From 2019 onwards, the maximum bias was less than 20 cm or 7% at every station. The model tends to overestimate extreme tidal high water at the German and Danish west coast (positive bias) in some years, but rather underestimates them in West and East Frisia (negative bias).

### Sea water salinity and temperature

**Fig. 7:**
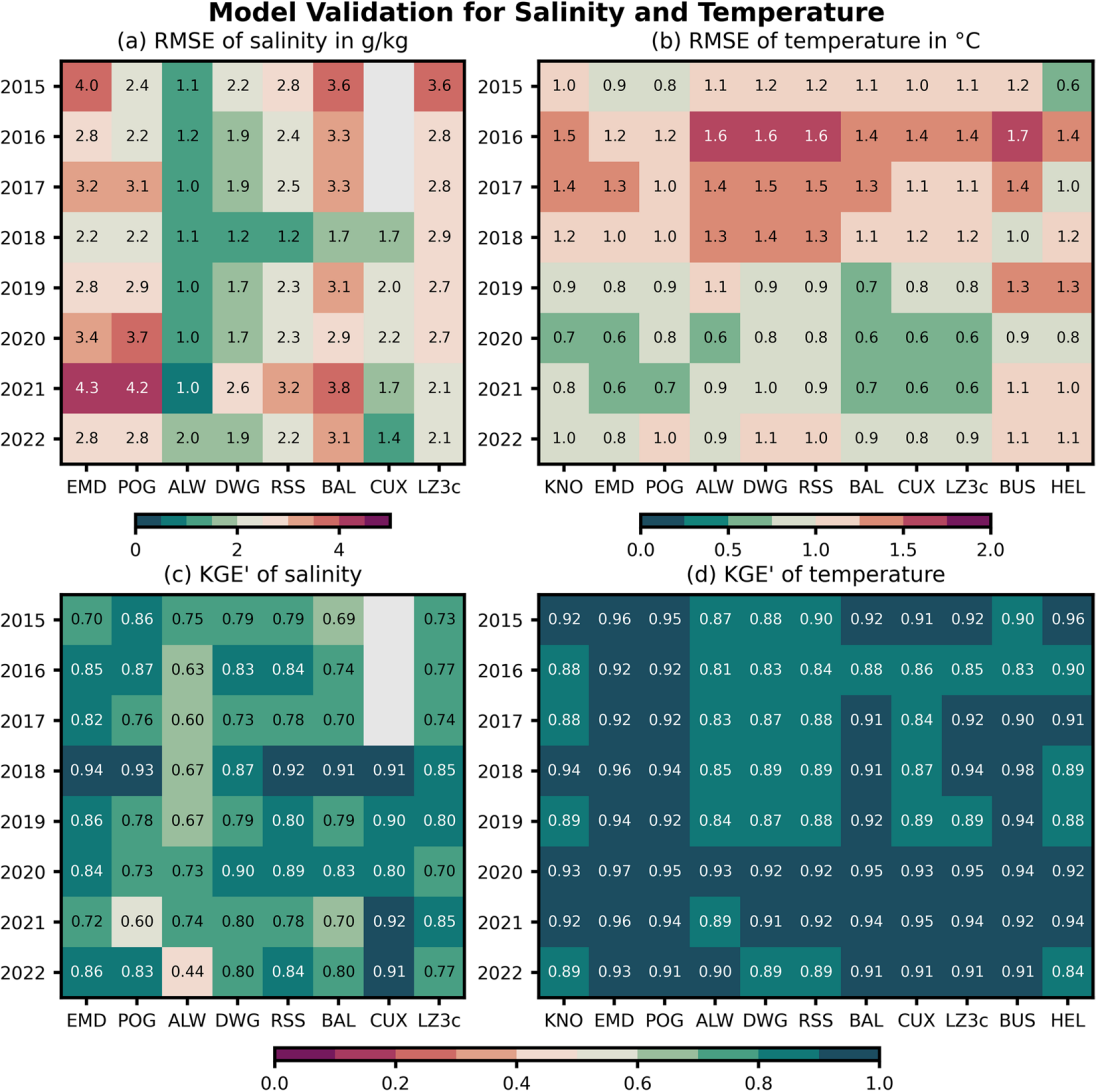
RMSE (**a,b**) and KGE’ (**c,d**) of sea water salinity (**a,c**) and temperature (**b,d**) from annual model-measurement comparisons. The model was evaluated at the grid cell and depth layer closest to the measurement locations. Salinity data at Cuxhaven (CUX) for 2015 to 2017 contain implausible measurements, which is why they were excluded from panel (a) and (c).

Salinity measurements were mainly carried out in the brackish waters of estuaries with only few offshore measurement stations. For sea water temperature, observations were available both near and off the coast. We used RMSE (Fig. 7a,b) and KGE’ (Fig. 7c,d) to conduct annual validations at the depth of the measurement device. Note that model deviation between observation and simulation was highest in the mixing zones of estuaries due to mixing processes and large salinity variance at these stations.

Sea water salinity validation indicated that the model reproduced the measurements with a RMSE mostly below 2.5 g/kg. Larger errors were generally confined to regions with strong salinity gradients in the mixing zones of the estuaries (Fig. 7a). Further offshore, the model performed better, e.g., the RMSE was only about 1 g/kg at ALW in the outer Weser estuary vs. 1.7 to 3.8 g/kg in the Weser’s mixing zone near BAL. KGE’ was almost always above 0.6 for salinity (Fig. 7c), indicating that temporal patterns were reproduced and that bias was low.

Comparisons of sea water temperature showed annual RMSEs between 0.6 and 1.7 °C in our model (Fig. 7b). The error decreased notably in the period of 2019 to 2022 compared to 2015 to 2018, indicating that the updated atmospheric forcing used from 2019 onward resulted in an improvement of the modeled sea water temperature. This is in line with the improved peak sea surface elevations resulting from more accurate wind forcing (i.e., Fig. 6c). KGE’ of the sea water temperature was always above 0.81, indicating very well-reproduced temporal patterns and only minor model bias (Fig. 7d).

### Waves

The spectral wave model was validated using a RMSE of the significant wave height (H_m0_), of the wave period second moment (T_m02_), and of the mean wave direction (Φ_m_) at five wave observation locations scattered in the project area (see Fig. 2). All gauges were wave rider buoys, except for the observational platform FINO1. As observational wave data were available less abundant and in lower quality than, e.g., sea surface elevations, fewer gauges were considered and the annual completeness of H_m0_ was indicated. In this context, completeness refers to the number of mutual time steps between model and observation with all model time steps being 100%. Gauges were chosen as a compromise between spatial coverage across the study site and maximum survey completeness. It should be noted that our model compares the mean wave direction during peak wave period against the observational data. This causes episodical offsets due to the lack of swell waves from the North Atlantic Ocean which our local model currently does not capture and causes high error residuals and therefore a higher RMSE in mean wave direction. No mean wave direction observations were available to the authors at the WA_SM wave rider buoy.

Wave validation in Table 4 indicates the presence of two groups: The western and southern comparisons (WA_SM to WA_ELB) displayed lower skill than their northeastern counterparts with a RMSE of more than 50 cm compared to less than 35 cm in H_m0_. The deviation from the measurement spreads evenly across the modeled years with RMSEs of 48 to 57 cm in WA_SM, 43 to 64 cm in WA_FINO1, 53 to 58 cm in WA_ELB, 24 to 30 cm in WA_SUEH, and 26 to 47 cm in WA_WES. The larger error in WA_WES in 2022 likely results from the poor observational data coverage of only 8% in that year.

**Table 4:** RMSE of the modeled significant wave height (H_m0_) and its annual completeness in percent, wave period (T_m02_), and mean wave direction (Φ_m_) at observational gauges.

Year	WA_SM			WA_FINO1			WA_ELB			WA_SUEH			WA_WES		
	H_m0_	T_m02_	Φ_m_	H_m0_	T_m02_	Φ_m_	H_m0_	T_m02_	Φ_m_	H_m0_	T_m02_	Φ_m_	H_m0_	T_m02_	Φ_m_
Unit	m (%)	s	deg	m (%)	s	deg	m (%)	s	deg	m (%)	s	deg	m (%)	s	deg
2015	0.51 (57)	1.4	—	0.61 (90)	1.0	54.0	0.58 (75)	1.0	55.9	0.30 (60)	0.7	28.4	0.33 (98)	1.2	35.4
2016	0.57 (80)	1.8	—	0.52 (72)	0.9	53.8	0.53 (39)	1.0	59.3	0.28 (95)	—	—	0.29 (98)	1.2	38.3
2017	0.57 (94)	1.6	—	0.64 (91)	1.1	51.0	0.58 (97)	1.0	55.7	0.28 (97)	0.7	28.4	0.30 (92)	1.0	29.3
2018	0.48 (99)	1.7	—	0.46 (67)	1.1	63.7	0.56 (96)	1.0	60.1	0.26 (81)	0.8	37.4	0.26 (88)	1.3	44.0
2019	0.50 (88)	1.7	—	0.43 (61)	0.9	52.1	0.55 (97)	1.0	58.1	0.24 (96)	0.8	30.0	0.30 (90)	1.1	34.4
2020	0.52 (92)	1.7	—	0.48 (81)	1.1	57.3	0.58 (90)	1.0	57.9	0.27 (100)	0.8	28.7	0.35 (47)	1.2	13.3
2021	0.54 (96)	1.9	—	0.57 (100)	1.2	56.3	0.56 (100)	1.0	56.4	0.26 (88)	0.8	31.2	0.28 (100)	1.3	34.6
2022	0.53 (82)	1.8	—	0.58 (93)	1.1	53.0	0.54 (100)	1.0	60.4	0.29 (36)	0.9	24,2	0.47 (8)	0.9	21.4

The completeness of H_m0_ indicates the number of trustworthy observational data. Where no data were available, a “-” was indicated.

**Fig. 8:**
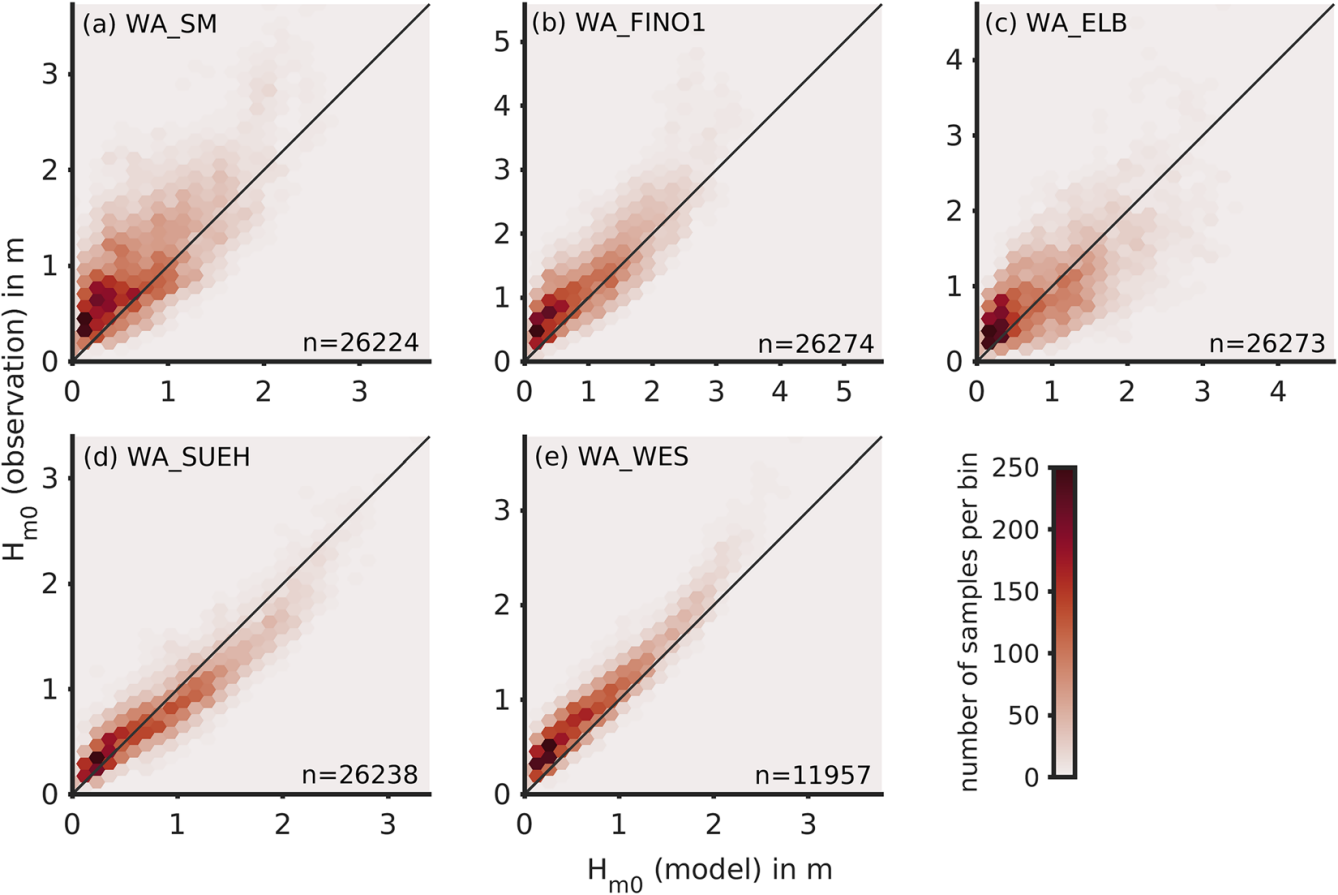
Comparison of the modeled and observed significant wave height (H_m0_) at observational sites (a-e) in the year 2021. Darker shades of red indicate a higher sample density. The maximum number of available samples (n) is given in the bottom right.

To put these numbers into perspective, a scatterplot between observed and modeled H_m0_ was created for the year 2021 at all locations from Table 4 (see Fig. 8). We find that the western and southern locations show a higher spread between model and observation although most samples seem to be located around the optimal fit with a tendency of wave height underestimation in the model. The model skill decreased at higher significant wave heights but low and moderate wave heights agreed well. This was reflected in the visual impression from time series (not included) where the visual pattern was reproduced, yet higher wave heights were underestimated. Thus, lower skill near high and peak wave heights are a limitation of our data even though it should be noted that measurement accuracy decreases in rough seas and other authors noted similar issues at peak wave heights when they used these observational data in model validation^60^.

### Sediment transport

**Fig. 9:**
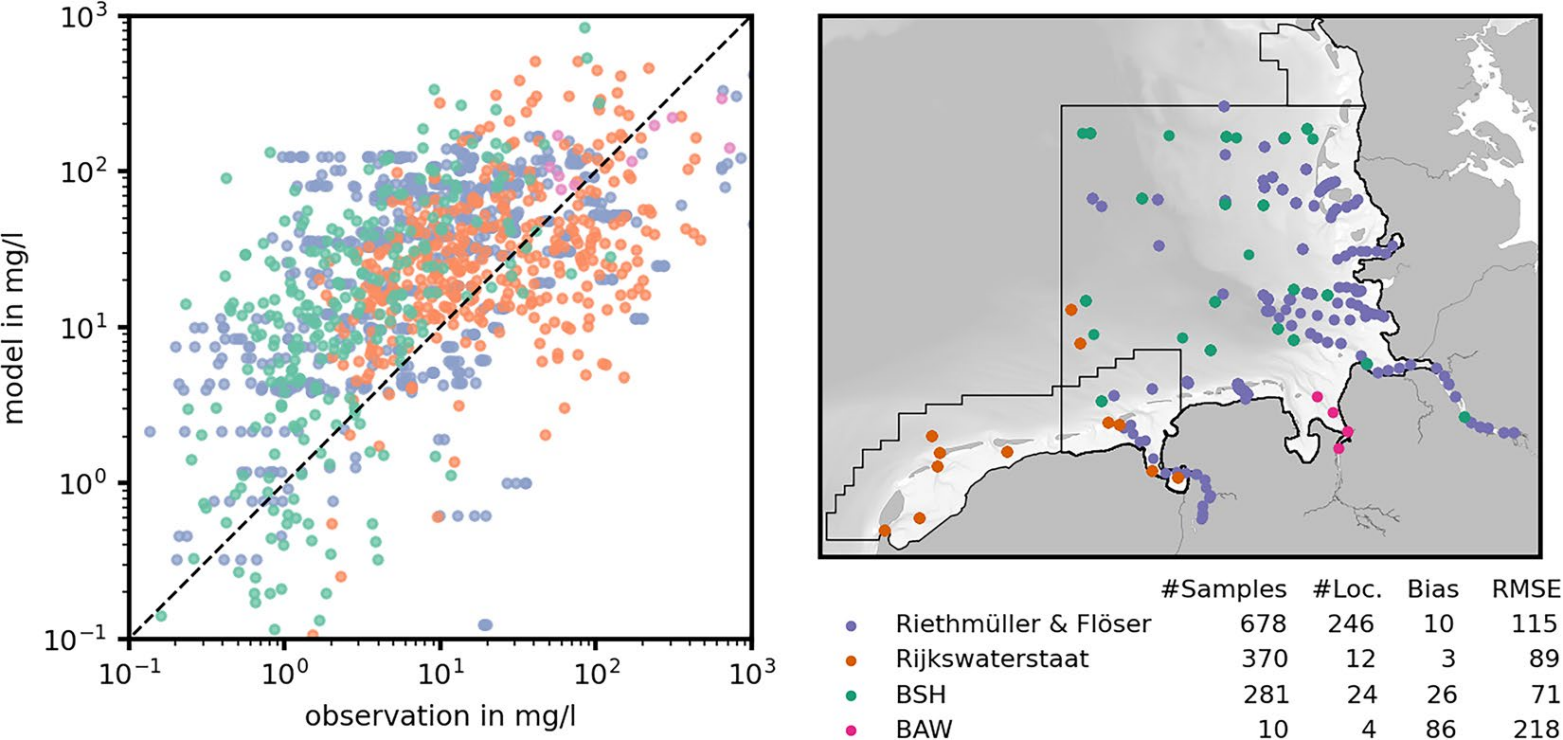
Validation of the modeled SSCs against observed, water sample-derived SSC from different data sources (**a**) and water sample locations (**b**). The table in the bottom right lists the number of samples, the number of locations as well as mean bias, and RMSE (both in mg/l) for all stations. Samples were separated by the data supplier.

**Fig. 10:**
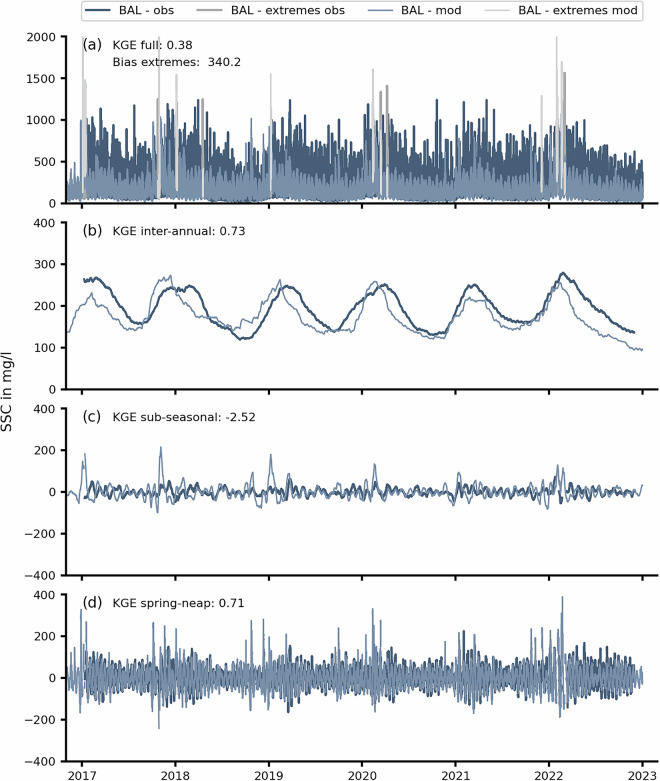
Decomposition of the SSC dynamics at the BAL gauge with observations in dark blue and model results in light blue. Extreme events (grey) are indicated based on a threshold of 1,243 mg/l. Model skill in the period of 2017 to 2023 is indicated by the adjusted Kling-Gupta-Efficiency (KGE’) and visualized on different time-scales for the full time series (**a**), the inter-annual variations (**b**), the sub-seasonal variations (**c**), and the spring-neap dynamics (**d**).

SSCs depend on sediment availability, large-scale processes, and local drivers. In a model, the consideration of several sediment fractions, grain-grain interaction, and transport parametrization introduces further complexity and thus uncertainty. We used a two-step process to validate modeled SSC in our model: First, we compared a large collection of water-sample-derived SSCs (high measurement accuracy) to validate the order of magnitude (Fig. 9). Second, we assessed time-dependent dynamics of turbidity-derived SSC time-series (low measurement accuracy) in the German Weser estuary on different timescales, i.e., a seasonal (inter-annual), a sub-seasonal (e.g., river discharge events), and a spring-neap time-scale (Fig. 10), to understand if the time series was reproduced. In the following, SSC refers only to inorganic components.

We used a total of 1,339 water samples at 286 locations in the data domain for the first step of SSC validation (Fig. 9). The modeled SSC was overestimated slightly within one order of magnitude and had an average bias between 3 and 26 mg/l. We considered the estimation within one order of magnitude as a success despite the uncertainty inherent to modeling suspended sediments and the potential presence of organics. Still, larger variations were evident near the mouths of the Weser, Elbe and the hyper-turbid Ems, both for very low concentrations offshore, and for several measurements with a wide concentration spread over short distances (Fig. 9a). The RMSE varied between different data sources, which we believed to result from a different spatial coverage. Higher RMSEs were evident for the BAW and Rithmüller and Flöser data, both of which often cover the more turbid estuaries.

In the second validation step, modeled SSC dynamics were compared to turbidity-derived SSC observations in the Weser estuary by decomposing the complete time-series into relevant time-scale constituents. We identified episodic storm surge SSC by flagging the highest sea surface heights, i.e., 99.99th percentile. As SSC concentrations remain high after a storm, bias was determined based on a relaxation period comprised of one day before and of two days after an event. Storm surge SSC was validated separately using a bias metric and was then excluded from subsequent time-series validation (Fig. 10a). Then, signals were decomposed by timescales (Fig. 10b–d) using a rolling window. The rolling window length decreased with each decomposition, and each cycle was subtracted by low to high frequency from the full signal to avoid double counting.

Our workflow is demonstrated at BAL (Fig. 10) which had the best agreement with the model. Comparing the full signal (a) indicated that the model usually underestimated observed SSC while episodic storm-surge peaks had a positive bias (overestimation) of 340 mg/l. Still, the KGE’ indicated fair agreement of 0.38 with low overall bias and a good representation of time-series characteristics. Inter-annual dynamics (Fig. 10b) were also captured well with a KGE’ of 0.73 and plausible visual agreement. As expected, higher SSCs were evident during the storm surge season in both the measured and modeled time series. The sub-seasonal variability (Fig. 10c), e.g., from river discharge events, demonstrated a worse KGE’ of −2.5 although the visual agreement could still be considered reasonable. We suspect that the overestimation of the model after storms weakens the correlation component of the KGE’ to a poor score. The spring-neap dynamics based on the tidal residual (Fig. 10d) indicated a KGE’ of 0.71 which stands in contrast to the supposedly poor score in the sub-seasonal timescale. This workflow demonstrates that the high uncertainty of the turbidity-derived SSC observations complicates a conventional validation assessment. We therefore recommend expert judgement and plausibility checks whenever our SSC data are applied.

Table 5 shows the KGE’ metric for additional gauges around the mixing zone of the Weser estuary. It becomes evident that the overall agreement is best within the mixing zone of the Weser and declines seaward to KGE’ values of approx. 0. Again, it must be noted that the KGE’ decreases outside of an estuarine mixing zone because the harmonic tidal signal in SSC declines, weakening the correlation within KGE’. Hence, all gauges demonstrate a fair inter-annual agreement and a weak score for sub-seasonal variability. The latter should be interpreted with care, considering the influence of SSC overestimation during extreme events. The agreement on the spring-neap time-scale varied between good (BAL), fair (DWG) and poor (RSS and NUF) with no obvious spatial correlation.

**Table 5:** SSC validation at observational turbidity gauges in the outer Weser estuary in the period of 2015 to 2022 for relevant different time scales. KGE’ > −0.41 indicates good model skill.

Time-scale	KGE’ (DWG)	KGE’ (RSS)	KGE’ (BAL)	KGE’ (NUF)
full	0.03	0.05	0.38	0.46
inter-annual	0.41	0.58	0.73	0.58
sub-seasonal	−18.7	−1.57	−2.52	−1.77
spring-neap	0.0	−0.7	0.71	−2.19

## Usage Notes

The development of a DTC was accompanied by a stakeholder participation process to ensure practical relevance and usability. Within these collaborations, we developed practical workflows, best-practice examples, and novel datasets with third-parties. All these efforts were documented and published as separate technical reports (in German) and linked to the relevant metadata of the guiding themes. Since several applications resulted in data publications, we found that use cases added transparency and demonstrated potential applications and limitations. This satisfies the bi-directionality component of a digital twin and partly contributed to interactive components. Examples are the development of a web-based parameter intersection tool^61^, a novel method of separating intertidal flats from supratidal marshes and subtidal channels by using topography in combination with modeled tidal high and low water^55^, or the computation of the cubed current velocity as estimator for tidal energy potential in the Wadden Sea^62^.

All data can either be downloaded directly from the repository of the BAW (https://datenrepository.baw.de/), via subsequent German or European data hubs, cited using a data-DOI, or be explored in an interactive web environment (https://mdi-dienste.baw.de/viewer/). To further add to the interactive component of a digital twin, we explored web-processing services and implemented them in this environment. Examples include the extraction of time-series from modeled data, dynamic bathymetry differences, web-based data cropping, or trajectory profile functionality. Furthermore, European and national additional layers such as national park boundaries or European bathymetry were incorporated as auxiliary layers. Finally, users may also upload their own local data or use other web-services.

## Data Availability

The hydrodynamic numerical model UnTRIM² (developed at the University of Trento, Italy), and its associated modules SediMorph and UnK as well as its postprocessor NCANALYSE (managed by the Federal Waterways Engineering and Research Institute, BAW, Germany) are not publicly available but underlying methods and concepts were published in the referenced literature of this paper. Harmonic analyses were conducted with the open-access MATLAB UTide functions^50^. Data product maps were generated with the open-access MATLAB package m_map^63^ and QGIS (version 3.40.8, last access 06 October 2025). KGE’ and its three components (r, γ, β) were computed with the python package hydroeval.

## Data availability

All published data can be obtained under https://datenrepository.baw.de/. Metadata entries were structured by the following guiding themes and can be found by adding the keyword “digital twin” to the search window or by following the digital object identifier (DOI) of the following citations (e.g., digital twin topography):

• topography^24^,

• intertidal topography^52^,

• surface sediments^27^,

• hydrodynamic data^53^,

• data analysis, e.g., tidal analysis^54^,

• and use cases^55^.

The web-viewing module can be viewed at https://mdi-dienste.baw.de/viewer/. All observational data in model validation were listed in the respective subsection of Methods. All model forcing data were listed in the subsections describing the models.
